# A mixed-methods study protocol on factors contributing to suicide clusters among Native American youth in a northern plains reservation

**DOI:** 10.3389/fpubh.2023.1281109

**Published:** 2024-01-08

**Authors:** Teresa Brockie, Michelle Kahn-John, Laura Mata Lopez, Eleesha Bell, Truth Brockie, Terry Brockie, Ellie Decker, Nancy Glass, Hannah Has Eagle, Kenneth Helgeson, Nona J. Main, Mina Kazemi, Reyna Perez-Monteau, Alicia Myrick, Katie E. Nelson, Adriann Ricker, Tammy Rider, Teeah Roberts, Deborah H. Wilson, Karen Yazzie, Nancy Perrin

**Affiliations:** ^1^Johns Hopkins School of Nursing, Baltimore, MD, United States; ^2^Fort Belknap Reservation Community, Agency, MT, United States; ^3^Montana Family Planning, Bozeman, MT, United States; ^4^Fort Belknap Tribal Health Department, Harlem, MT, United States; ^5^Auckland University of Technology, Auckland, New Zealand

**Keywords:** suicide cluster, Native American, youth, reservation, protective factors, risk factors, mixed-methods

## Abstract

**Introduction:**

Suicide and suicide clusters within Native American Reservation communities are devastating to the entire community and increase individuals’ risk for suicide over the lifespan. The objective of this paper is to describe the Indigenous community-based participatory research protocol implemented in partnership with the Fort Belknap Indian Community in Montana, United States. The study protocol was developed to understand suicide risk and protective factors, and community-derived solutions, in a reservation community with history of a suicide cluster and high rates of youth suicide.

**Methods:**

In this mixed-methods study, qualitative data from youth, adults, and service providers and quantitative data from 200 adolescents and young adults (aged 14–24 years) were collected in Fort Belknap, Montana from May – December of 2022. Qualitative data were collected first via in-depth interviews and focus groups. Survey questions included validated and pre-tested measures of factors youth experience across socio-ecological levels. Thematic analysis was applied to the qualitative data; and logistic regression models were used to examine relationships within the quantitative data.

**Discussion:**

This study will add a multi-dimensional perspective to our current understanding of (1) risk and protective factors for suicide, community-derived postvention solutions, and insights on community assets, and (2) the current health and psychosocial status of youth in the Fort Belknap community. This study may serve as an exemplar of co-created, culturally safe solutions designed to address mental health resource gaps. Next steps include development of a suicide crisis response tool kit and a culturally aligned postvention intervention that will enhance individual, family, and community survivance.

## 1 Introduction

Native Americans (inclusive of American Indians and Alaska Natives in the United States; more broadly referred to as Indigenous Peoples, globally) have been exposed, collectively and historically, to an array of traumatic experiences stemming from settler colonialist systems. Actions involved attempts to systematically erase Indigenous peoples and culture through forced displacement from traditional and sacred land bases, depopulation through starvation, military action, forced boarding school experiences and family separation (1–6). The impact of these historical exposures is long-lasting and intergenerational (7, 8). Decades of research has shown that Native American communities experience profound distress and health inequities, including higher rates of mortality and suicide (9–11). Suicide has been a leading cause of death for Native American youth aged 15–24 years for over 40 years (12–14), and this population has the highest rate of death by suicide of any ethno-racial group in the United States (US) (15). Particular communities bear the burden of suicide, including suicide point clusters. In general, little is known about why suicide clusters form—and at times persist. Research regarding suicide clusters among Native Americans shows increased risk among rural reservations and Alaska villages (16, 17). Native American youth aged 10–24 years accounted for approximately 35% of all deaths by suicide in the Native American community (14). A suicide cluster is defined as a group of suicides, suicide attempts, or self-harm events that occur closer together in time and space than would normally be expected in a community. While rare, suicide clusters tend to occur in remote and tight-knit communities (such as reservations), where mental health resources are scarce and unemployment and economic hardships are common (18). The increased number of deaths and fear of additional losses associated with suicide clusters can have a profound impact on Native American families and communities. It can also exacerbate collective and intergenerational trauma (19). Literature has documented the stark disparities in suicide-related deaths among reservation-based Native American youth compared to their non-Native American counterparts (10). However, there remains a limited understanding of differences that exist across Native American communities and tribes, particularly in the context of existing community-based suicide response systems, assets, and infrastructures (18, 19). Among the subgroup of Native Americans (2.2 million) who receive their healthcare through the Indian Health Service (IHS)—described as “having the worst health outcomes” of any ethnic group in the US—there are vast differences in suicides (20). Among the 12 IHS Areas, suicide rates are highest among those in the Alaska Area, at 38.9 per 100,000 people, a rate 3.6 times the US national rate of 10.9 and 7 times higher than the California Area, which possesses a rate of 5.5 per 100,000. Suicide is highest among remote IHS Areas, underscoring the impact social determinants of health have on suicide, and that suicide is not race related (21).

Native American reservations, established by treaty during the Reservation Era (1851–1887) following the enactment of the Indian Appropriations Act, are areas of concentrated poverty, high distress, and increased morbidity and mortality (10, 22, 23). The risk of suicide among reservation-based Native American youth is nearly six times higher compared to those living in other areas (13). Reservations, such as Fort Belknap, experience concentrated poverty and segregation, which limits access to resources and contributes to increased morbidity and mortality (24–26). Furthermore, the persistent underfunding of IHS, coupled with the failure to allocate resources based upon need, has created limited access to resources and networks required to meet residents’ full potential, including challenges accessing health care and mental health resources (27). The gap in access to mental health care and culturally appropriate services has further exacerbated the suicide crisis across reservation-based Native American communities (10, 27–29).

As of 2020, the state of Montana had the third highest suicide mortality rate in the US, with a crude rate of 26.1 deaths per 100,000 – ranking among the top five states with the highest suicide mortality rates for over 30 years (30). Between 2011 and 2020, the nine Tribes located in Montana had the highest rate of suicide, with a crude rate of 32.0 deaths per 100,000, despite comprising only 6% of the total state population (30, 31). The Fort Belknap Reservation is extremely remote and has an average population density of <4 persons per square mile, compared to the national average of 94 persons per square mile (32). Fort Belknap also ranks among the poorest areas in Montana and in the US with approximately 50% of children under the age of 18 years living in poverty (33). In terms of educational attainment, <25% of Fort Belknap residents hold college degrees (33).

Despite these alarming disparities, Fort Belknap demonstrates considerable community support and mastery during crises, potentially resulting from strong family and community ties, connection to culture, language, and land. These distinct social and cultural assets can promote psychological resilience and collective well-being and foster psychosocial resources that can protect Native American communities against suicide and suicide clusters (25, 34, 35). For example, studies have shown interconnectedness among Native American families, systems, and communities can enhance available networks and sources of social support, thereby buffering the risk of suicide – even in the presence of potent community risk factors (36–38). Native American communities across North America are also leading culture and language revitalization efforts to reclaim traditional knowledge and practices that were formally eradicated through centuries of cultural genocide – promoting connection to tribal and cultural identity, and spiritual and traditional practices (39, 40). Increasingly, Native American communities are nurturing these cultural strengths to develop community-driven responses and interventions to address and prevent suicide (39, 41, 42).

### 1.1 Objective

This paper outlines the protocol for an ongoing study to understand suicide risk and protective factors, and community-derived solutions in a reservation community. With three main components, the study aims are to: (Aim 1) Explore environmental, cultural, spiritual, and relational factors (e.g., opportunities for belonging, connectedness) in the Fort Belknap community to understand how they contribute to, or protect against, youth suicide via qualitative focus groups and interviews; (Aim 2) Identify risk and protective factors for youth suicide at the individual, familial, communal, and societal levels among Aaniiih and Nakoda youth aged 14–24 years using survey methodology; and (Aim 3) Identify resources for mitigating suicides and natural helpers for the development of accessible, relevant, and effective suicide postventions on the Fort Belknap reservation through cultural and community asset mapping.

## 2 Methods and analysis

### 2.1 Conceptual model

**Figure 1:**
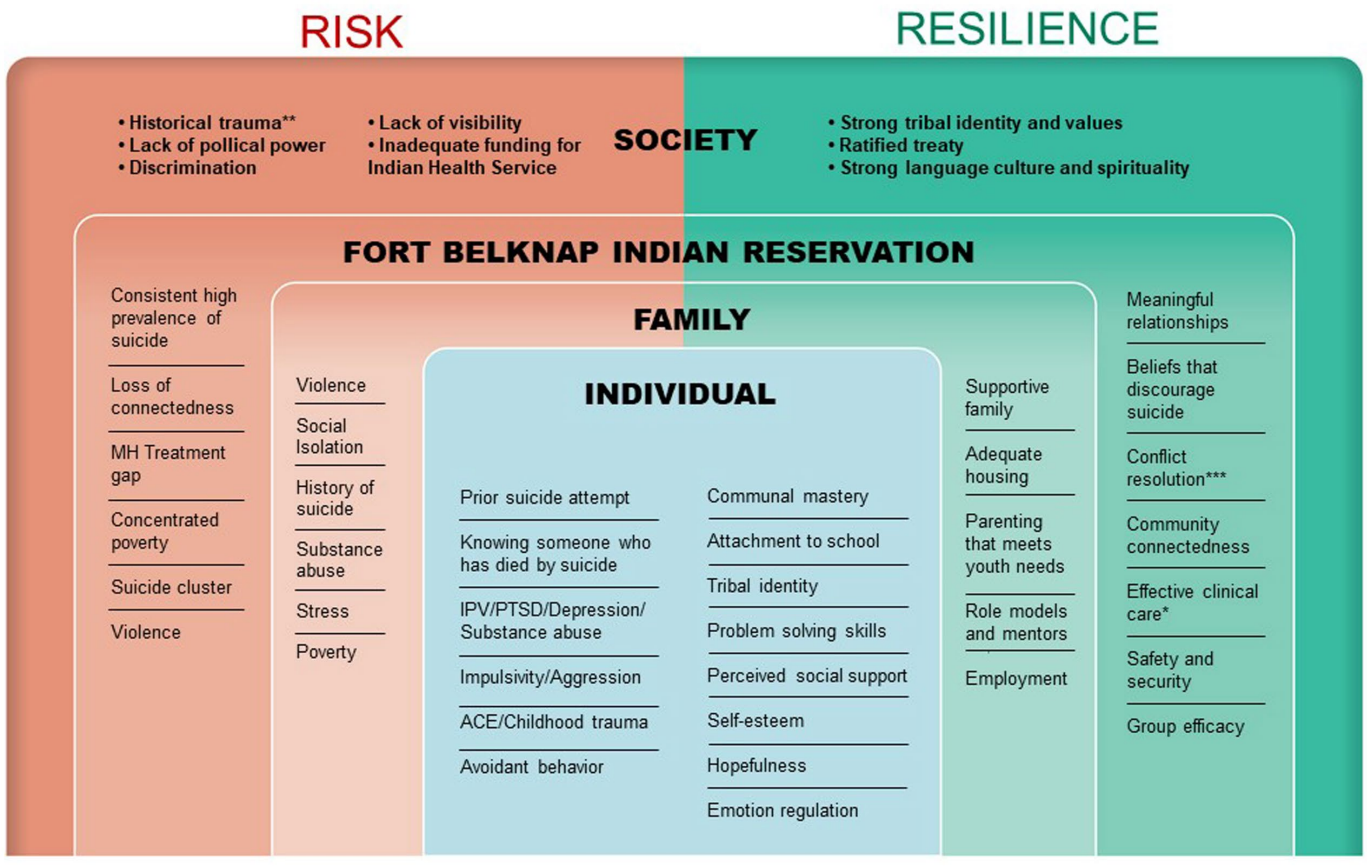
An adapted conceptual model of multilevel factors associated with risk of and protection for reservation-based youth suicide.

The conceptual model (Figure 1) that guides this work was adapted from the *Social-Ecological Model: A Framework for Prevention* (43), in collaboration with the study’s Tribal Advisory Board (TAB) and informed by lessons learned from over 10 years of work with the neighboring Fort Peck Assiniboine and Sioux Reservation (11). Rooted in socio-ecological principles, the model encompasses two key concepts: (a) There are *multiple levels* of individual, relationship, community, and societal factors that contribute to suicide and thus, it is necessary to intervene at multiple levels; and (b) *reciprocal causation*—individual behaviors shape and are shaped by the social environment. The model guided questions asked in Aim 1 and the selection of measures for Aim 2.

### 2.2 Study design

We used a sequential exploratory mixed-methods design, which was implemented for the purpose of triangulation and corroboration of research findings. In alignment with this design, the qualitative focus groups and individual interviews were conducted for Aim 1. Preliminary analysis of the qualitative data informed development, refinement, and selection of the subsequent quantitative survey components. A 251-item questionnaire, including demographics and 17 measures was utilized to collect data from an estimated 200 Aaniiih and Nakoda youth aged 14–24 years. Analysis of the qualitative and quantitative data was conducted sequentially and independently to explore consistency and/or divergence across findings. Following completion of both qualitative and quantitative research, two points of integration will have occurred: (1) *a priori* (aligned with survey guide) flexible coding and index analysis informed design and selection of the quantitative measures; and (2) during phase two of qualitative inductive thematic analysis (primary indexing and secondary thematic) confirmed findings of the quantitative results while offering thematic insights on experiences of the community.

### 2.3 Setting

**Figure 2:**
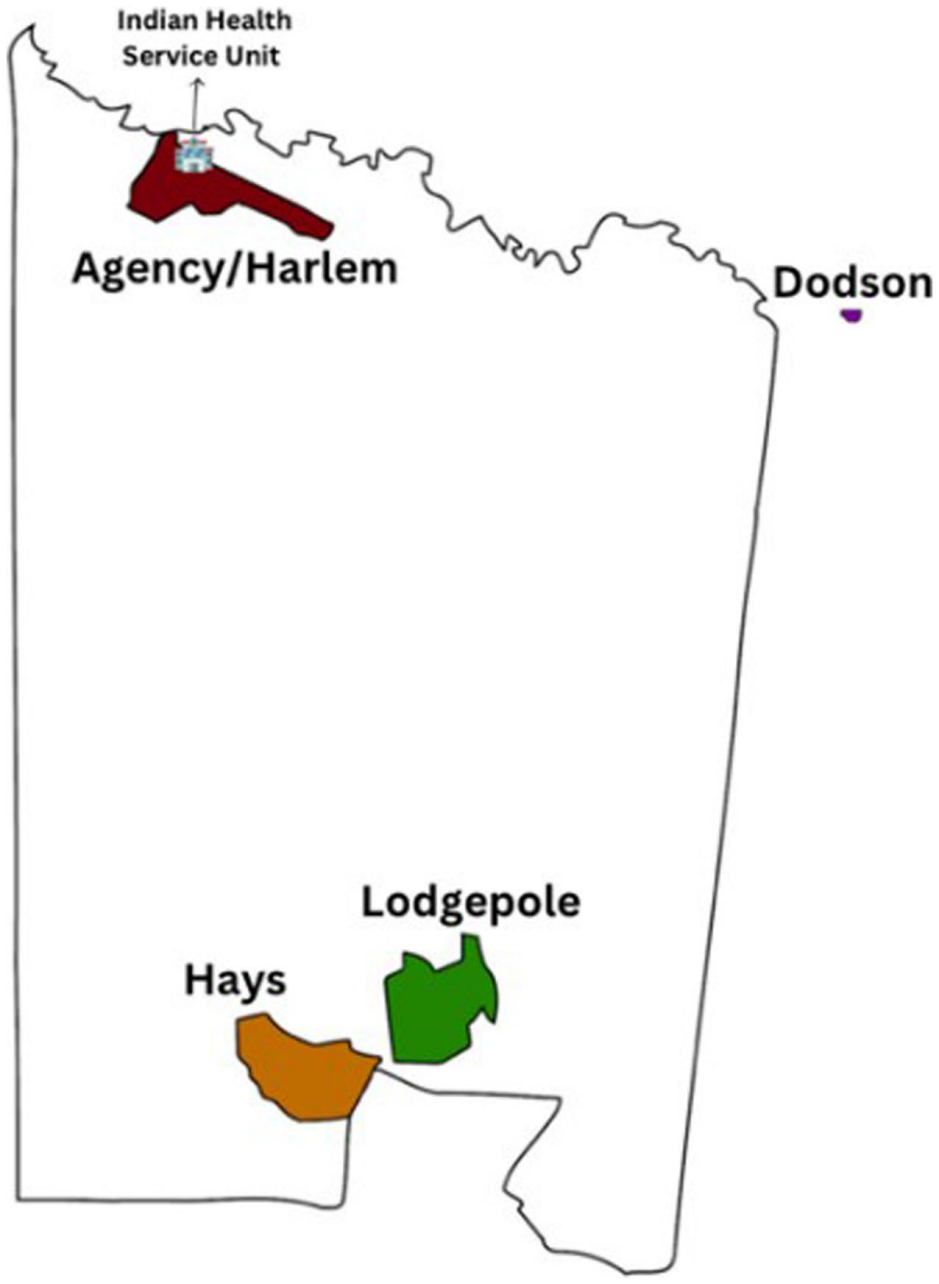
Map of Fort Belknap Reservation.

The Fort Belknap Indian Reservation was established by treaty in 1885 and is governed by the elected Fort Belknap Community Council. The Fort Belknap Reservation is part of the Billings IHS Area, which services 11 Tribal Nations that encompass one reservation in Wyoming and seven reservations in Montana. Fort Belknap is in northcentral Montana and spans 675,147 acres (about half the area of Delaware) and is home to approximately 7,000 enrolled Aaniiih and Nakoda members (44, 45). There are four distinct communities in Fort Belknap (Hays, Lodgepole, Agency/Harlem, and Dodson), each with varying strengths, resources, and community characteristics (Figure 2). The IHS Service Unit provides health services to the Fort Belknap community by treaty and is in Fort Belknap Agency, Montana (44).

In 2019, Tribal leaders declared a state of emergency following three youth deaths by suicide in 1 month (26). The increasing urgency to address youth suicide as a public health crisis led to the development of a research-practice partnership between the Fort Belknap Tribes, Aaniiih Nakoda College (ANC), and the Johns Hopkins University (JHU) School of Nursing in 2020.

### 2.4 Community-based participatory research approach

Our study leverages Indigenous and community-based participatory research methodologies to center partnerships and capacity building, which is critical to promoting evidence-based advocacy and health equity for Indigenous communities (23, 46–48). The Fort Belknap-JHU partnership encompasses partners from the Tribal Health Department and Public Health Nursing Department, who are a powerfully engaged team of public health nurse leaders and advocates who address the public health needs of the community while prioritizing a culturally aligned health care delivery model. Another essential partner is ANC, one of 35 tribal colleges in the U.S., whereby education is grounded in Indigenous ways and knowledge (49).

A critical step in establishing trusting partnerships with tribal communities includes the formation of a Tribal Advisory Board (TAB) (50). Our TAB served as the voice of the community, providing input in study design and recruitment (identification of key stakeholders and community leaders), qualitative interview guide development, selection of quantitative measures, data interpretation, and dissemination. This level of engagement directly infuses the voices of community members into the research process and strengthens the relevance and impact of study findings. Team members also participated in traditional ceremonies with community members, which served to establish and strengthen relationships, appropriately acknowledged the importance of data collection, and supported our collective healing given the sensitive nature of the study topic. Additionally, we engaged local leaders who work with youth in the community, hereafter referred to as “community champions,” to plan, promote, and support research efforts. The in-depth processes utilized to develop and leverage our relationships with the Fort Belknap community will be reported in a future publication.

### 2.5 Recruitment

Recruitment for our focus groups, in-depth interviews, and survey were initiated by the local community research coordinator in collaboration with community champions. We recruited participants in all four communities through word-of-mouth, flyers, and radio public service announcements. For asset mapping (Aim 3), we will recruit participants who have been involved in suicide intervention and prevention programming and research through established networks. The Fort Belknap community and TAB were also engaged in providing recommendations for participant recruitment. Utilizing snowball sampling, we asked participants to identify additional contacts for potential participation. Community champions facilitated community engagement activities such as sharing recruitment materials at local events, booking meeting facilities, and gathering food, supplies and equipment, highlighting the importance of involving community. Table 1 depicts inclusion and exclusion criteria for the study. Living within a 1-h drive of the Fort Belknap IHS Unit was a component of our safety protocol to ensure participants had adequate access to care and/or could be referred for follow-up as needed following study participation.

**Table 1:** Inclusion criteria.

*Aim 1: Qualitative*
Willingness to participate Currently work or have worked in systems of care for individuals with suicide risk or have lost a loved one to suicide or be a service provider ≥14 years of age Parent/guardian consent and adolescent assent for participants <18 years of age Currently live within 1-h drive of Fort Belknap IHS Unit Identify as a member Fort Belknap Community
*Aim 2: Quantitative survey (including pilot)*
Willingness to participate Currently live within 1-h drive of Fort Belknap IHS Unit 14–24 years of age Parent/guardian consent and adolescent assent for participants <18 years of age Identify as a member of Fort Belknap Community
*Aim 3: Community and cultural asset mapping*
Willingness to participate TAB members, Spiritual leaders in the community, or have worked in suicide intervention or prevention programming ≥ 18 years of age

### 2.6 Participant incentives and debriefing

At the end of each data collection segment all participants received an honorarium for their time and knowledge contributions. Participant incentives were as follows: Aim 1 and survey pilot participants received a $40 Visa gift card; Aim 2 participants received $30 cash. Additionally, all participants received a local mental health resource card and smudge was offered after all study activities for culturally appropriate debrief spiritual centering, cleansing, and calming (48, 51) Every Aim 2 participant was engaged in cultural debrief and mental health screening, with referrals provided if indicated, upon survey completion.

### 2.7 Aim 1

#### 2.7.1 Sample size

Sample size centered on maximizing inclusion of diverse perspectives from across the four Fort Belknap communities (52, 53). Sample sizes were estimated using the following parameters: community size, suicide risk level, and TAB feedback. We drew on guidance from content and methodological experts to determine number of focus groups and their make-up (i.e., 5–10 participants per group, with separate groups for youth, adults, and service providers) (54).

#### 2.7.2 Data collection

We conducted theoretically grounded, semi-structured focus groups and individual interviews with a diverse sample of community stakeholders and members impacted by suicide (*N* = 79). We offered individual interviews in the event participants felt uncomfortable in a focus group setting, given the sensitive nature of the topic. Data collection occurred in-person on the Fort Belknap Reservation—in adherence with local COVID-19 safety protocols (e.g., room capacity limits, temperature checks, masks, etc.). Participants completed a brief anonymous demographic questionnaire before their session. Interviews and focus groups were audio recorded, professionally transcribed, de-identified, and reviewed by the research team for accuracy. We used an in-depth interview guide, which was developed based on feedback and input from our TAB, subject matter experts, and community members.

#### 2.7.3 Analysis

In phase one of the qualitative analysis, index analysis was conducted by performing flexible coding in f4Analyse^®^ (version 2.5.2), a qualitative analysis software. The index analysis was framed by the survey guides and allowed the team of reviewers to become familiar with the data. The team coded and indexed data into *a priori* index code categories (risk factors, protective factors, grief experience, and postvention solutions) (53, 55). The COREQ criteria were used for structuring our analysis (56). The indexed data will be further analyzed to generate meaningful themes in phase two of qualitative analysis. The resulting themes—and the relationships among them—will be harnessed for designing a postvention response and reported in a separate manuscript.

### 2.8 Aim 2

#### 2.8.1 Sample size

We used data from the neighboring Fort Peck Reservation to estimate the prevalence of suicide (7). Based on this data, we estimated that 45% of participants would screen positive for lifetime suicidal ideation and 35% would report a lifetime suicide attempt. Our goal was to detect odds ratios of approximately 1.50 or greater for each one unit increase in the predictor variable. With statistical power = 0.80 and alpha = 0.05, we can detect significant OR > 1.49 for suicide ideation and OR > 1.52 for attempts with a sample size of 200. As such, recruitment targets were 75 participants from both Hays and Agency/Harlem, and 25 each from the Lodge Pole and Dodson communities, respectively, to reach this total.

#### 2.8.2 Data collection

Survey pilot: Prior to implementation, the survey was piloted with 11 participants to ensure participant acceptability, usability, and cultural relevance. Participants provided informed consent and for those <18 years, parental consent and participant assent were achieved. Each individual completed the survey via tablet in REDCap, a HIPPA-compliant data management software, or via paper-based copy. Participants then completed an individual interview about their experience completing the survey. Interviews were audio recorded, and trained interviewers also took detailed notes.

Survey implementation: For survey administration, the study team collaborated with community champions across the four communities to schedule data collection sites and dates, which took place in schools and community centers. Cell phone usage was not allowed in the computer labs being used for survey conduction and participants were seated at every other computer for safety and privacy purposes. These protocols were implemented as part of our Standard Operating Procedures to promote overall fidelity for data collection and reduce the potential for social desirability bias. Paper copies of the survey were available if needed to troubleshoot any technology issues, though the need for this ultimately did not arise.

#### 2.8.3 Measures

Quantitative data were collected using pre-tested and psychometrically validated questionnaires that have previously been used with Native American populations and/or validated in cross-cultural settings (Table 2). As noted above, measures were revised based on Aim 1 preliminary findings, survey pilot findings, and feedback from TAB members and experts in youth suicide research. At a later point, three measures (polysubstance use, traumatic grief, and racial discrimination) were added at the request of the TAB and content experts so were not pilot tested with Fort Belknap youth. Aligning with our conceptual model, the 17 measures are grouped into four domains: individual, relationship/family, community, and society and are described in depth below.

**Table 2:** Measures.

Measure	Description	# Items
*Individual*		
Orthogonal Cultural Identification Scale	This scale was modified based on pilot feedback to measure Aaniiih and Nakoda tribal identity using a 4-point Likert scale (ranging from 0 = “None” to 3 = “A lot”) (57, 58).	6
Center for Epidemiologic Studies Depression Scale-Revised (CESD-R-10)	Depression symptoms were assessed using this scale, which used a 4-point Likert scale (ranging from 0 = “Rarely or none of the time” to 3 = All the time”). The total score is the sum of all responses, with a cut-off score of greater than eight indicating clinically significant symptoms (59).	10
Benevolent Childhood Experiences	Positive childhood experiences (between birth to age 18) were measured using this scale, which used dichotomous (“yes” or “no”) items (60).	10
Adapted Columbia Suicide Severity Rating Scale (CSSR-S) (Primary outcome)	Suicide risk (based on severity of ideation, intensity of ideation, behavior, and lethality) was measured using this scale, which used dichotomous (“yes” or “no”) items (61).	5
The Schutte Self Report Emotional Intelligence (SSEIT)	Emotional intelligence was measured using this scale, which used a 5-point Likert-scale (ranging from 0 = “Strongly disagree” to 4 = “Strongly agree”) (62).	33
Short Form – PTSD Checklist for DSM-5 (PCL-5)	Trauma symptoms were measured using this scale, which used 5-point Likert scale items (ranging from 0 = Not at all” to 4 = “Extremely”) (63).	4
Cyberbullying Victimization Scale	The frequency of cyberbullying experiences was measured using this scale, which used a 5-point Likert scale (ranging from 0 = “Not at all” to 4 = “Very often”) (64).	27
Philadelphia Adverse Childhood Experience (ACE) Questionnaire	Adverse childhood experiences occurring in the first 18 years of life were measured using this scale, which used dichotomous (“yes” or “no”) and Likert scale items capturing adverse childhood experiences (65).	22
CDC Youth Risk Behavior Surveillance (YRBS) Survey	The substance use portion of the YRBS questionnaire was adapted to assess first use, lifetime, and current use of substances and binge drinking. The scale uses a combination of dichotomous (“Yes” or “No”) and multiple-choice items to capture the age of onset of substance use, quantity, and frequency of use (66).	27
Polysubstance use	This scale was adapted to assess both alcohol and fentanyl use with other substances over the past 12 months using a combination of dichotomous (“yes” or “no”) and 3-point Likert scale items (ranging from 0 = “never” to 2 = “always”) (67).	4
Racial discrimination	Scale developed to assess discrimination experienced by Native American individuals (74)using a 3-point Likert scale (ranging from 0 = “Never” to 2 = “Always”) (68).	11
*Relationship/family*		
Brief Family Relationship Scale (BFRS)	The BFRS was utilized to assess family relationship domains of Cohesion, Expressiveness, and Conflict subscales (9 items each) using 3-point Likert scale items (ranging from 0 = “Not at all” to 2 = “A lot”). Subscales were developed with Native American youth (69).	25
Traumatic Grief Inventory Self Report	This questionnaire was used to assess traumatic grief experiences and losses using 5-point Likert scale items (0 = “Never” to 4 = “Always”) (70).	19
Witness to Intimate Partner Violence (IPV)	Witness to IPV was assessed with two dichotomous (“Yes” or “No”) items, “In your whole life, have you ever seen your mother get hit, slapped, punched, or beaten up?” and “In your whole life, have you been hit, slapped, kicked or otherwise physically hurt?” (71).	2
*Community – fort Belknap Indian Reservation*		
Communal Mastery Scale	Communal mastery was assessed with this scale, which was developed from two commonly employed measures of mastery and self-efficacy and adapted for more collectivist communities including Native American populations (34, 72) and Alaskan Native youth (73). The measure used 4-point Likert scale items (ranging from 0 = “Strongly disagree” to 3 = “Strongly agree”).	10
*Society*		
Historical Trauma	Historical Trauma was assessed utilizing two dichotomous (“Yes” or “No”) and an additional question evaluating the impact of these experiences (“Negative,” “Positive,” “Neutral,” “Do not know,” or “Refuse to Answer”). These questions were developed from our previous work (74).	3
Historical Loss	This scale quantifies the types of losses that Native American tribes might have experienced in the past and how often they are thought about using 6-point Likert scale items (ranging from 0 = “Never” to 5 = “Several times a day”) (68, 69).	12

##### 2.8.3.1 Primary outcome

The primary outcome of interest for this study was lifetime suicidal ideation and attempts. Suicide risk was measured with five dichotomous Yes/No items from the *Columbia Suicide Severity Rating Scale (CSSR-S),* which has been used widely to identify suicide risk among diverse populations (61). Items included were those that assessed suicide risk based on severity of ideation, intensity of ideation, behavior, and lethality.

##### 2.8.3.2 Individual level variables

Tribal identity was assessed with an adapted version of the *Orthogonal Cultural Identification Scale* (57). This 6-item scale uses a 4-point response scale (None to A Lot) and was modified based on pilot feedback to measure cultural identification with Aaniiih and Nakoda tribal identity. The original version of this scale was utilized with Indigenous youth (α = 0.90) (58). Depression symptoms were assessed with the 10-item *Center for Epidemiologic Studies Depression Scale-Revised (CESD-R-10),* which uses a 4-point response scale (Rarely or None of the time to All of the time) (59). The CESD-R-10 is an abbreviated version of the CESD which has been validated among Native American populations (75, 76). Positive childhood experiences (between birth to age 18), including perceived feelings of safety, support, and predictability, were measured using 10-items, with dichotomous (yes or no) response options from the *Benevolent Childhood Experiences Scale* (60). Emotional intelligence was measured by the 33-item *Schutte Self-Report Emotional Intelligence Test (SSEIT)* with a 5-point response scale (Strongly disagree to Strongly agree) that includes four subscales: emotion perception, utilizing emotions, managing self-relevant emotions, and managing others’ emotions (62). Trauma symptoms were measured by the 4-item *Short Form – Post-traumatic Stress Disorder (PTSD) Checklist* for Diagnostic and Statistical Manual of Mental Disorders DSM-5 (PCL-5) with a 5-point response scale (Not at all to Extremely) (63). Cyberbullying experiences, and frequency of experiences, were measured by 27 items on a 5-point response scale (Not at all to Very often) from the *Cyberbullying Victimization Scale* (64). Adverse childhood experiences occurring in the first 18 years of life and related to the community, family, and the individual were measured by 22 items from the *Philadelphia Adverse Childhood Experience (ACE) Questionnaire* (65). This scale uses a combination of dichotomous (Yes/No) and Likert scale items capturing adverse childhood experiences. High-risk substance use, first use, lifetime, and current use of substances and binge drinking were assessed with selected questions from the *CDC Youth Risk Behavior Surveillance (YRBS) Survey* (66). Additionally, polysubstance use, or the concurrent use of substances over the past 12 months was assessed using an adapted version of Midanik et al. (67) polysubstance use scale. Perceived discrimination was measured with the 11-item *Racial Discrimination Scale* with a 3-point response scale (Never, A few times, Always), which was originally developed to assess discrimination experienced by Native Americans (7, 68).

##### 2.8.3.3 Relationship/family level variables

Family relationships were assessed using 25 items with a 3-point response scale (Not at all, Somewhat, A lot) from the *Brief Family Relationship Scale (BFRS):* The BFRS was adapted from the 27-item Relationship dimension of the Family Environment Scale (77) and was utilized to assess family relationship domains of Cohesion, Expressiveness, and Conflict subscales (9 items each). Subscales were developed with Native American youth (69). Traumatic grief was assessed using the 19 items from the *Traumatic Grief Inventory Self Report (TGI-SR)*: the first part of the questionnaire asks about the death of the loved one and the second part asks about symptoms related to loss of a loved one, using a 5-point Likert scale (“Never” to “Always”) (70). The first part was removed after pilot testing. Lifetime exposure to domestic violence was assessed using one item: “In your whole life, have you ever seen your mother get hit, slapped, punched, or beaten up?” and lifetime victimization to domestic violence using one item: “In your whole life, have you been hit, slapped, kicked or otherwise physically hurt?,” using dichotomous (Yes or No) responses (7, 71).

##### 2.8.3.4 Community level variables

Communal mastery was assessed with the 10-item *Communal Mastery Scale* with a 4-point response scale (Strongly Disagree to Strongly Agree) (34). The scale was developed from two commonly employed measures of mastery and self-efficacy and adapted for more collectivist communities including Native American populations (34, 72) and Alaskan Native youth (73).

##### 2.8.3.5 Societal level variables

Historical trauma was measured using three Yes/No questions developed from our previous work (78): (1) “That you know of, did any of your great-great-grandparents or elders attend mandatory boarding school?” (2) “Was their attendance and experience in boarding school talked about in your home?” and (3) “In your opinion, was their experience in mandatory boarding school negative, positive, or neutral?” (74). Historical loss was measured by the 12-item *Historical Loss Scale* with a 6-point response scale (Several times a day to Never) (79). This scale was used to quantify the types of losses that Native Americans might have experienced in the past and how often they are thought about in the present (79). This scale uses a 6-point Likert scale (“Several times a day” to “Never”) to measure losses and has been used in our previous study (α = 0.93) (74).

#### 2.8.4 Analysis

All variables were summarized descriptively (means, standard deviations, interquartile range, and percentages) and distributions examined to determine if they met the assumptions of the underlying analytical models. The pattern of missing data was examined to determine if it was missing at random. Multiple imputations were used to handle missing data at the scale level. Model building was conducted by examining the bivariate relationship between each of the predictor variables and suicide attempt/ideation using logistic regression. Variables significant at the 0.05 level were moved into a multivariable logistic regression model and the area under the curve (AUC), sensitivity, and specificity was estimated to determine model fit. Next, a multinomial model was estimated with three levels of the outcome (no ideation/attempt, ideation without an attempt, suicide attempt). Suicide ideation without an attempt was the reference category utilized to explore the factors that may distinguish between suicide ideation and attempt. We began using a bivariate model and moved significant variables forward to the multivariable model. Results were also graphically displayed using forest plots. Findings from this analysis will be reported in a separate manuscript.

### 2.9 Aim 3

#### 2.9.1 Sample size

We will recruit 10–12 individuals from the Fort Belknap community with expertise in suicide prevention and intervention via our existing networks to participate in roundtable discussions.

#### 2.9.2 Data collection

We will utilize a roundtable discussion format to conduct asset mapping with community experts to identify natural helpers and resources to inform development of postvention interventions and a suicide cluster response plan. Community Asset Mapping consists of six steps: (1) defining community boundaries; (2) identifying and involving partners; (3) determining what type of assets/resources are available; (4) generating lists of organizations, associations, and institutions in the community; (5) generating lists of individuals in the community; and (6) organizing a visual map or diagram about where these resources, organizations and individuals are and how they communicate with each other (80). Together with our TAB, community leaders, and stakeholders, we will develop a Fort Belknap Community Crisis Management Plan for Preventing and Responding to Suicide Clusters. This plan will present reigning national recommendations, promising practices, and guidelines for community response. Collaborative consultation with reservation stakeholders in the design of such a plan will ensure maximum community and cultural validity for the locally developed postvention plans.

#### 2.9.3 Analysis

The data-derived themes from Aim 1 will be particularly vital to this analysis, highlighting participant perspectives, cultural insights, and culturally aligned approaches—therein identifying community assets. Integration and comparison of qualitative (Aim 1) and quantitative (Aim 2) results will allow for triangulation to confirm areas of consistency or convergence. The resulting themes will be summarized in written form and circulated to the TAB and other community stakeholders for comments and recommendations for further improvement. Once finalized, the blueprint of the resulting community-driven postvention response will be passed along to tribal leaders for consideration of implementation and further planning.

## 3 Quality control and safety procedures

The study team performed internal quality management of study conduct and data collection, storage, documentation, and completion. All research staff were trained in and followed the Standard Operating Procedures to ensure study fidelity and participant safety. Quality control procedures were implemented as outlined below.

### 3.1 Informed consent

Study staff completed a review of completed consent form documents to ensure signature, date, time, and any optional participation notes (such as future contact) were correctly completed. The Program Coordinator reviewed each form and provided feedback to the study team to ensure proper consenting procedures were followed.

### 3.2 Recruitment report

The Program Coordinator produced a report each week to share at study progress meetings. This included: number of active participants, eligible individuals who required follow-up, conflicts of interest, number of participants who enrolled/declined/consented/undecided, and the number of focus groups/interviews/surveys completed overall.

### 3.3 Team training

The Program Coordinator facilitated training for all data collection team members on the Standard Operating Procedures and strategies for collecting data with safety and validity at the forefront. Expert trainers provided team trainings on the following topics: Qualitative Data Collection: Recruitment, Asking, Listening and Ethical Considerations; Keeper of Stories: The Responsibility of Storytellers in an Indigenous Research Context; Psychiatric Diagnostic Criteria and Cultural Safety; Working with Youth; QPR (Question, Persuade, Refer) Suicide Prevention; Safety Planning and Mandated Reporting, REDCap Basics; and Minimizing Bias in Quantitative Data.

### 3.4 Data management and storage

Audio recordings and transcripts were saved to the JHU OneDrive platform. Survey data were stored in REDCap (81). Hard copy documents were stored both in double locked file cabinets at the JHU School of Nursing office and in a standardized filing system in OneDrive. Survey data were reviewed at regular meetings by the Data Manager to ensure participant responses were correctly captured. All records will be kept in a secure location for a minimum of 7 years and to age 23 for pediatric participants, in accordance with Johns Hopkins Medicine Record Retention policy. Only key study team members (PI, study coordinator, and on-site coordinator) have access to the master list of participant names and IDs.

### 3.5 Participant safety and referrals

At completion of each survey, participants were flagged if their assessment scores indicated clinical levels of depression (a CESD-R score of 10 or above), moderate active trauma symptoms (a PCL-5 score of 8 or above), and/or suicide risk (any “yes” response to question number 1, 3 and 4 in the Adapted CSSR-S scale). A trained study staff member with a nursing background performed a debrief during which clinical flags were reviewed with each participant. In addition, referrals to local mental health providers were offered and participants were provided a mental health and referral resource card. Cultural debriefing and smudge were also offered before and after data collection to maximize participants’ safety, comfort, and healing.

## 4 Discussion

This study will provide multi-dimensional perspectives on the risk and protective factors for youth suicide and community-derived postvention solutions grounded in community assets and strengths. It will also enhance our understanding of the current health and psychosocial status of youth in the Fort Belknap community. Findings may be useful for addressing behavioral health needs for reservation communities across Indian Country. The US Government’s lack of resource investment and failure to address the physical and psychosocial health needs of Native Americans living on reservations established by treaty demonstrates little progress has been (and is likely to be) made towards developing context-specific suicide interventions in these communities. The variations in suicidal activity, risk factors, and mental health access across the 12 IHS geographical areas indicate tribal differences that require distinct exploration and tailored co-creation of culturally informed and community-derived interventions. To eliminate geographic disparities in suicide-related deaths and suicide clusters impacting reservation-based Native American communities, it is vital to focus research and resource allocation on Native American communities that experience disproportionately high suicide rates. In addition, the complexity of compounded trauma (individual, familial, community, historical, and intergenerational), the mental health treatment gap, and psychosocial stressors experienced by underfunded reservation communities require a multi-faceted, culturally aligned approach that prioritizes equity of voice and power between the research team and the community.

The contribution of this work will showcase the ongoing need to prioritize finding solutions to address the disproportionate mental health comorbidities (depression, anxiety, substance use disorder, complex trauma, and grief) in Fort Belknap. This work will inform a suicide crisis response tool kit and the development of meaningful culturally aligned, and safe solutions and interventions that will enhance individual, family, and community survivance (82). Overall, our study will serve as an exemplar of co-created, culturally safe solutions designed to address mental health resource gaps, which may be useful for other research-practice partnership teams.

## 5 Ethics and dissemination

This study was authorized by Fort Belknap Tribal Resolution #182–2020 and received Human Subjects approval from the Johns Hopkins School of Medicine (00305879) and Aaniiih Nakoda College Institutional Review Boards (IRBs). Protocol amendments were submitted as an amendment to both IRBs. All manuscripts and presentations are submitted to ANC IRB for review and approval. We obtained a Certificate of Confidentiality (CoC) through the National Institutes of Health (NIH), which further protects the privacy of study participants. Due to the CoC, investigators and others who have access to research records will not disclose identifying information except when the participant consents or in certain instances when federal, state, or local law or regulation requires disclosure. NIH expects investigators to inform research participants of the protections and limits to protections provided by a Certificate issued through this Policy.

## Ethics statement

The studies involving humans were approved by Fort Belknap Tribal Resolution #182-2020 and received human subjects approval from Johns Hopkins School of Medicine (00305879) and Aaniiih Nakoda College Institutional Review Boards. The studies were conducted in accordance with the local legislation and institutional requirements. Written informed consent for participation in this study was provided by the participant if 18 years of age or older; if under 18, participants provided assent and participants’ legal guardians/next of kin” provided written consent.

## Author contributions

TBr: Conceptualization, Funding acquisition, Investigation, Methodology, Resources, Supervision, Writing – original draft, Writing – review & editing. MK-J: Formal analysis, Investigation, Methodology, Writing – original draft, Writing – review & editing. LM: Investigation, Writing – original draft, Writing – review & editing. EB: Investigation, Writing – review & editing. TrB: Investigation, Writing – review & editing. TeB: Writing – review & editing. ED: Project administration, Supervision, Writing – original draft, Writing – review & editing. NG: Conceptualization, Formal analysis, Funding acquisition, Methodology, Writing – review & editing. HH: Validation, Writing – review & editing. KH: Validation, Writing – review & editing. NM: Investigation, Methodology, Writing – review & editing. MK: Investigation, Methodology, Project administration, Writing – original draft, Writing – review & editing. RP-M: Validation, Writing – review & editing. AM: Investigation, Project administration, Validation, Writing – review & editing. KN: Investigation, Writing – original draft, Writing – review & editing. AR: Conceptualization, Investigation, Methodology, Writing – review & editing. TaR: Funding acquisition, Resources, Writing – review & editing. TeR: Investigation, Writing – review & editing. DW: Investigation, Writing – original draft, Writing – review & editing. KY: Funding acquisition, Resources, Writing – review & editing. NP: Formal analysis, Investigation, Methodology, Writing – original draft, Writing – review & editing.
